# Arthroscopy-Assisted Core Decompression and Bone Grafting for Avascular Necrosis of the Hip

**DOI:** 10.1016/j.eats.2024.103127

**Published:** 2024-07-17

**Authors:** Tyler R. Mange, Christen E. Chalmers, Dean Wang

**Affiliations:** a Department of Orthopaedic Surgery, University of California Irvine, Orange, California, U.S.A; b Department of Biomedical Engineering, University of California Irvine, Irvine, California, U.S.A

## Abstract

Although specific techniques vary, core decompression is generally accepted as the treatment of choice for precollapse avascular necrosis (AVN) of the hip to delay or prevent progression of the disease. This can be combined with hip arthroscopy to allow visual assessment of the femoral head as well as treatment of intra-articular pathologies, which may contribute to pain and joint degeneration. We describe a technique of hip arthroscopy and concurrent core decompression using an expandable reamer and bone grafting for treatment of hip AVN. This allows for minimally invasive treatment of both bony and intra-articular soft tissue pathologies, which are often concomitantly present in hip AVN disease, while minimizing reaming of healthy femoral bone.

Avascular necrosis (AVN), or osteonecrosis, of the hip remains a challenging problem to treat in orthopaedic surgery. The overall prevalence is increasing, particularly as a sequela of systemic corticosteroid treatment for COVID-19, and advanced disease predictably results in femoral head collapse and arthritis. Hip AVN accounts for about 10% of all total hip arthroplasties performed in the United States.^1^ In early stage, precollapse AVN of the hip, treatment remains controversial and includes bisphosphonate medication, core decompression with or without bone grafting, and vascularized fibular grafting, with no therapies currently proven to reliably stop progression of the disease. Core decompression with adjunctive bone grafting is one technique for precollapse AVN and is generally accepted as superior to nonoperative treatment.^1^, ^2^, ^3^ Some authors have proposed combining core decompression with hip arthroscopy to address concomitant intra-articular pathology such as acetabular labral tears and/or femoroacetabular impingement lesions, as well as to ensure no intra-articular bone graft extravasation after the core decompression.^4^, ^5^, ^6^, ^7^, ^8^ Acetabular labral tears have been reported to be prevalent in approximately 64% of hips with precollapse AVN.^9^

When performing a hip core decompression, a major concern is increased fracture risk with large-diameter decompression.^1^^,^^7^ An alternative is drilling of multiple small holes, which may not provide similar core decompression results.^1^^,^^7^^,^^10^ In this report, we describe a technique for arthroscopy-assisted core decompression with autologous and allogeneic bone grafting using an expandable reamer, which allows for effective decompression without creation of a large-diameter core (Video 1, Table 1).

**Table 1:** Pearls and Pitfalls of the Described Surgical Technique

Pearls	Pitfalls
Gradually withdraw the Jamshidi needle while aspirating to maximize collection of progenitor cells from the endosteal surfaces	Avoid penetration into joint when drilling the femoral head, which can lead to bone graft extravasation and unwanted subchondral collapse
Use of a postless table allows for easy transition between arthroscopy (on traction) and core decompression and bone grafting/harvest (off traction), followed by arthroscopy (on traction)	Excessive antegrade reaming in the femoral neck region can result in stress risers and increase the risk of postoperative femoral neck fracture
Impact drill guide into the lateral femoral cortex to maintain trajectory into the femoral head	Expanding the reamer too quickly while reaming can cause significant torque on the handle attachment, causing it to break
Start with the smallest diameter reamer and gradually expand to fully ream all necrotic bone and avoid breaking the expandable reamer at its handle	Inadequate closure of interportal capsulotomy can lead to microinstability and worse outcomes
Arthroscopic visualization in the joint after core decompression ensures no extravasation of bone graft or iatrogenic subchondral collapse on the femoral head	

## Surgical Technique

### Step 1: Preoperative Workup

Patients presenting to our clinic with hip pain undergo routine history and physical examination and radiographic imaging, including standing anteroposterior views of the pelvis and affected hip(s), Dunn lateral views, and false profile views. If not already performed, magnetic resonance imaging of the hip is obtained to evaluate for intra-articular pathologies. The patient is counseled on their diagnosis, treatment options, and the risks, benefits, and postoperative course after surgery.

### Step 2: Operating Room Setup and Patient Positioning

**Fig 1:**
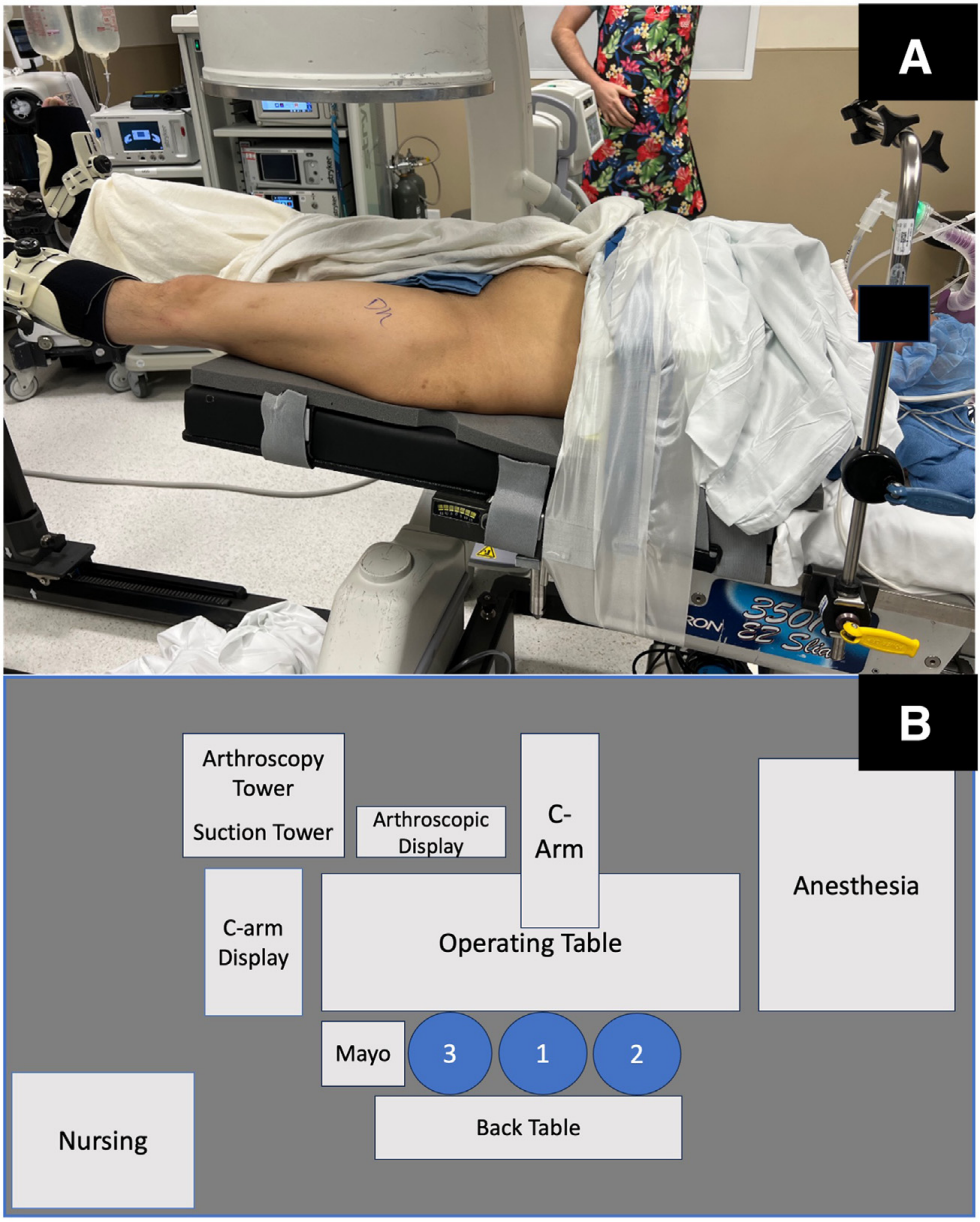
(A) Layout of operating room with the patient positioned supine on the postless traction table for a left hip procedure. (B) Schematic of the operating room layout for a left hip procedure (1: surgeon, 2: assist, 3: surgical tech).

The patient is brought to the operating room, induced under spinal or general anesthesia, and then transferred to the postless distraction operating table (Guardian; Stryker) in the supine position. The operative room layout is represented in Figure 1. The feet can be placed into the corresponding liners and boots prior to the patient being induced. Care is taken to position the patient with the greater trochanters just distal to the notches in the specialized foam padding to avoid issues with obtaining intraoperative fluoroscopy images. The boots are secured to the table and the arms are crossed over the patient’s chest and secured. The C-arm is brought in from the nonoperative side with the monitor placed at the feet, and fluoroscopic images are obtained prior to draping to ensure adequate imaging and traction.

### Step 3: Bone Marrow Aspiration

**Fig 2:**
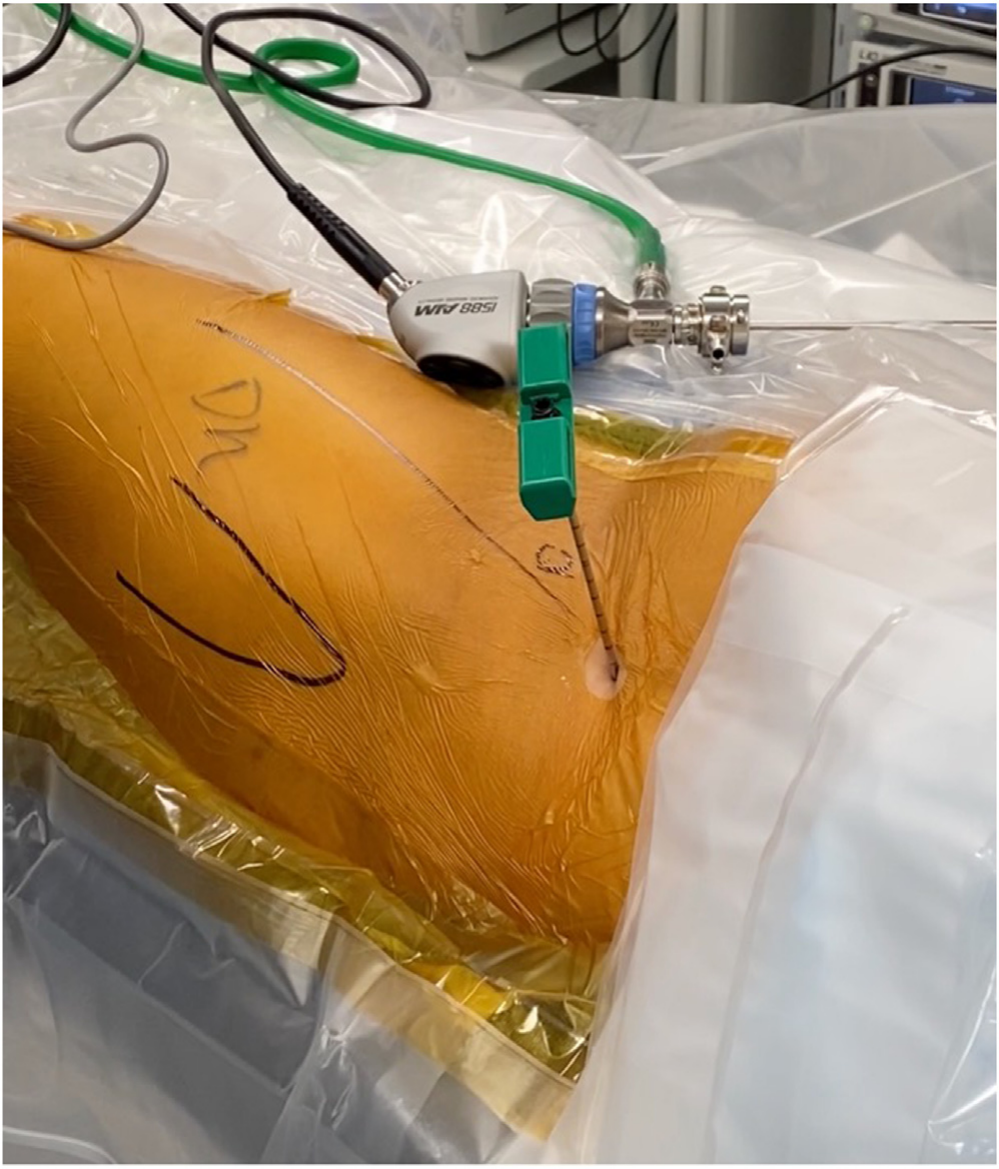
Prior to beginning the arthroscopy, a Jamshidi needle (green handle) is advanced into the left iliac crest for bone marrow aspiration. The aspirate is then concentrated using a commercial centrifuge.

**Fig 3:**
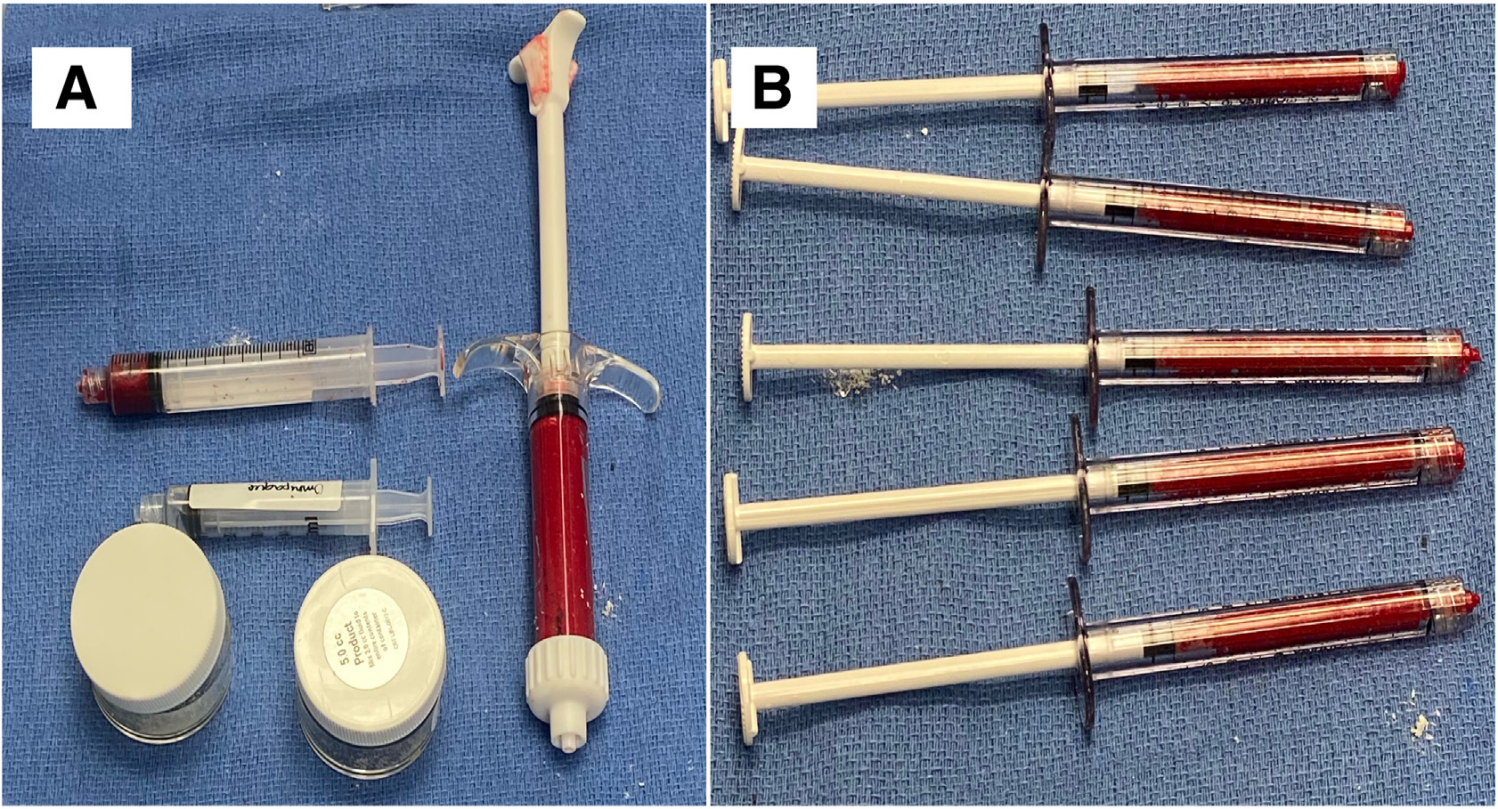
(A) 10 cc of AlloSync (Arthrex; black arrows), 4 cc of bone marrow aspirate concentrate (white arrow), and 1 cc of iohexol contrast media (red arrow) are combined into a large syringe (right). (B) The mixture is then loaded into the small syringes compatible with the delivery cannula.

After prepping and draping from the iliac crest to the distal thigh, the anterior iliac crest is identified and the Jamshidi needle (Arthrex) is used to pierce through the skin. After percutaneously feeling the inner and outer tables of the iliac crest, the needle is then positioned centrally with an appropriate trajectory, and a mallet is used to perforate into the cancellous bone. Next, the needle tip is swapped for a blunt tip and advanced further into the cancellous bone. With 8 cc of anticoagulant citrate dextrose, solution A preloaded in the syringes, at least 50 cc of bone marrow aspirate is then obtained while slowly withdrawing and rotating the needle to effectively collect the progenitor cells on the endosteal surfaces (Fig 2). This is then concentrated to about 4 cc using a commercial centrifuge (Angel; Arthrex) and combined with 10 cc of injectable demineralized bone matrix allograft (AlloSync; Arthrex) and 1 cc of iohexol contrast media on the back table (Fig 3). This wound can be dressed with a simple dry dressing.

### Step 4: Diagnostic Arthroscopy and Labral Repair

**Fig 4:**
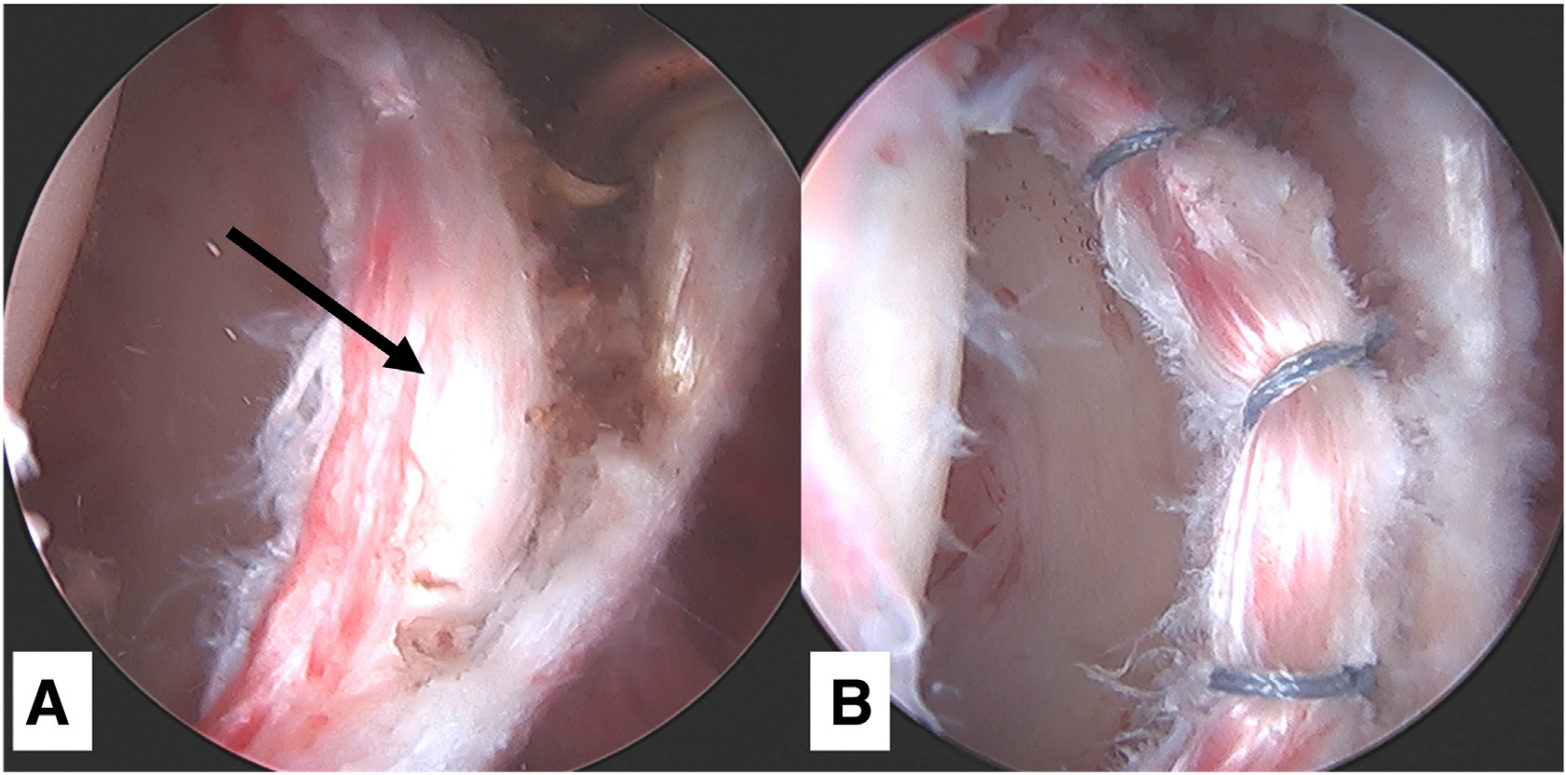
Viewing from the mid-anterior portal in a left hip, (A) labral tear (black arrow) noted on diagnostic arthroscopy with the patient supine and traction applied, and (B) Labrum repaired with knotless anchors.

Distraction is applied to the hip joint. Under fluoroscopic guidance, a standard anterolateral portal is then created, and a 70° arthroscope is introduced. A mid-anterior portal is created, and a diagnostic arthroscopy is performed, including assessment of the femoral and acetabular cartilage. The femoral head is probed to evaluate for any areas of articular cartilage softening or subchondral collapse. If any intra-articular pathology such as a labral tear requires treatment, an interportal capsulotomy is performed. For labrum repairs, the proximal capsule is elevated off the acetabular rim, and the subspine region and pincer lesions are decompressed. A distal anterolateral portal is then established for insertion of knotless all-suture anchors (FiberTak; Arthrex) on the anterosuperior acetabular rim, and the labrum is repaired according to the surgeon’s preference (Fig 4).

### Step 5: Core Decompression

**Fig 5:**
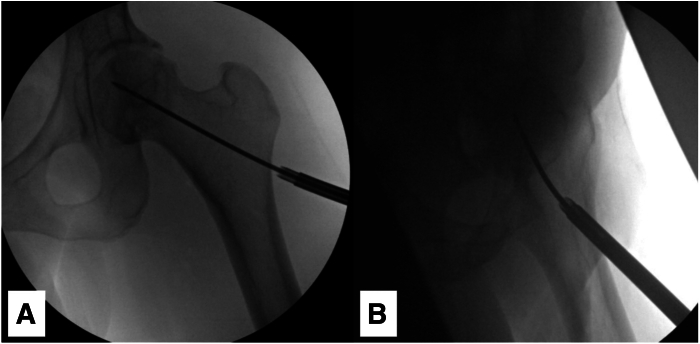
With the left hip off traction, the 2.4-mm guidewire for the expandable reamer system is advanced into an area of osteonecrosis in the left femoral head using anteroposterior (A) and lateral (B) C-arm guidance.

**Fig 6:**
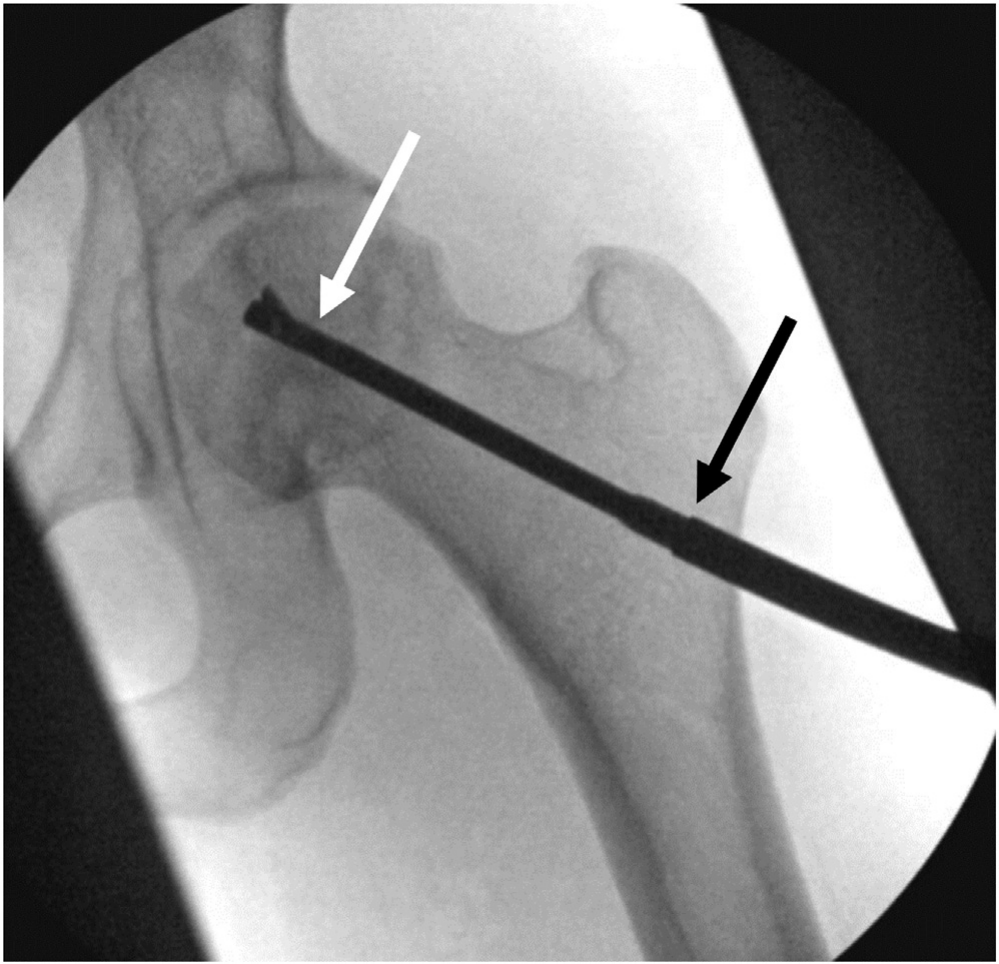
With the drill sleeves impacted into the lateral aspect of the proximal femur to maintain appropriate trajectory (black arrow), the expandable reamer (white arrow) is advanced into the femoral head by hand and used to gradually ream necrotic bone up to 18 mm in diameter. Care is taken to avoid reaming bone within the femoral neck.

After completion of the arthroscopy and any treatment of intra-articular pathologies, traction is then released, and the arthroscopic instruments are removed, leaving in place the radiolucent cannulas. Under fluoroscopic guidance, the site over the vastus ridge and proximal lateral femur for the entry point of the core decompression is localized, and a 2-cm incision is made through the skin, subcutaneous tissue, and iliotibial band. A Cobb elevator is used to expose the proximal lateral femur. A drill guide and sleeve from the AVN expandable reamer system (Arthrex) is positioned flush on the lateral femoral cortex at an appropriate starting point, and a 2.4-mm guidewire is inserted with appropriate trajectory toward the apex or anterosuperior region of the femoral head under fluoroscopic guidance and bulb saline irrigation (Fig 5). The senior author (D.W.) prefers to insert the guidewire freehand, with the arthroscopic drill guide handle mainly used as a handle to position and maintain the drill sleeve on the lateral femoral cortex. The drill sleeves (5 and 2.4 mm) are impacted 10 mm into the lateral cortex to maintain the entry point and trajectory, and the 2.4-mm drill sleeve is removed. The 5-mm reamer is then placed over the guidewire and reamed to the same depth as the guidewire. The reamer and guide pin are then removed, and the expandable reamer is manually inserted through the 5-mm tract into the femoral head. The reamer is then manually deployed and expanded, starting at the lowest diameter (6 mm) and gradually expanded to its maximum diameter (18 mm), turning clockwise and counterclockwise to ream through the necrotic bone until low resistance is felt (Fig 6). Once the maximum diameter is achieved, the reamer is withdrawn distally in an antegrade fashion to the level of the femoral head-neck junction while manually reaming to create a small cavity in the femoral head. Care is taken to avoid reaming within the femoral neck, an area that does not contain necrotic bone and can be predisposed to stress risers with excessive reaming. The expandable reamer is then docked and withdrawn from the hip.

### Step 6: Graft Placement

**Fig 7:**
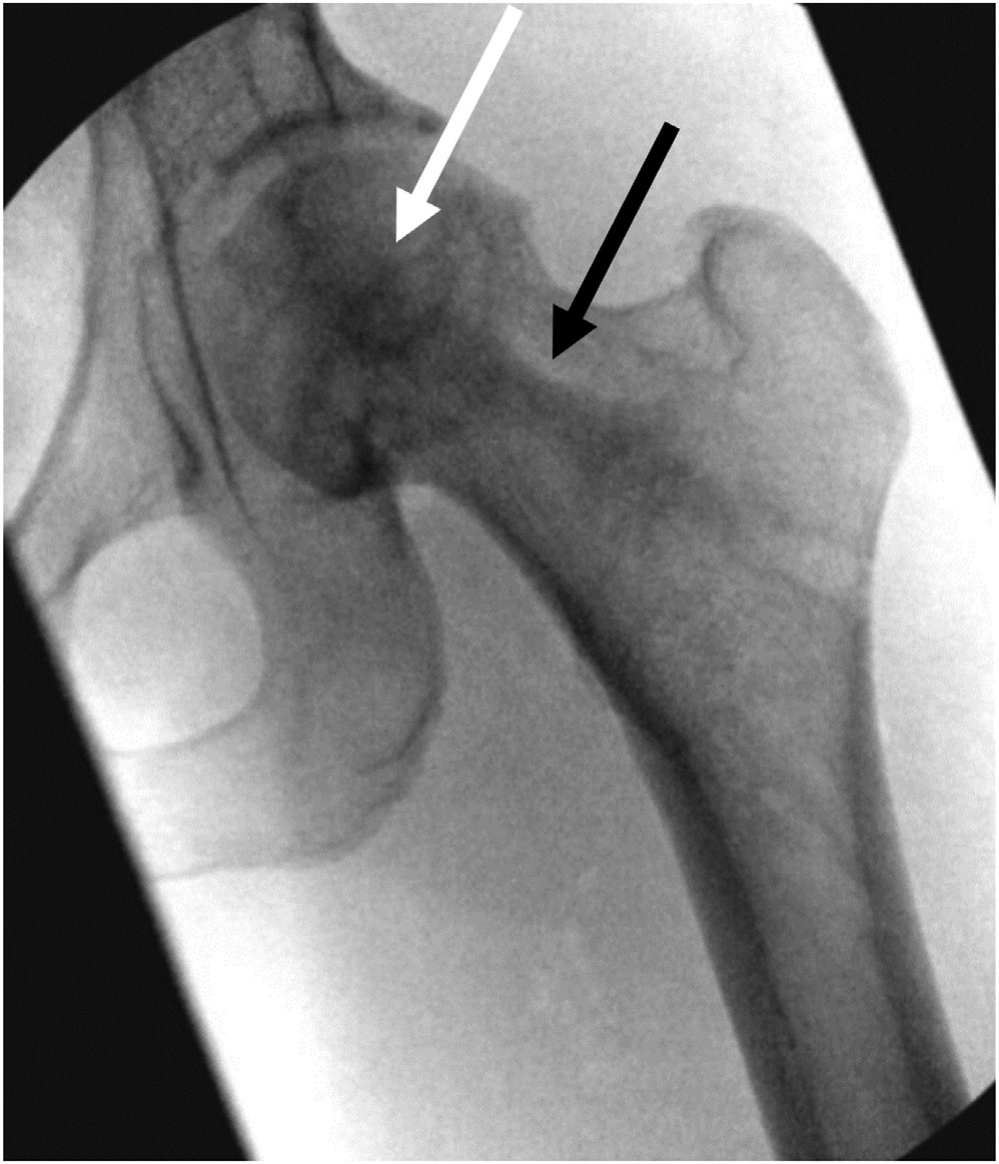
Final radiographs of the left hip demonstrating completed core decompression and bone grafting. Graft is placed in the decompressed area of the femoral head (white arrow) as well as along the reamer path (black arrow).

**Fig 8:**
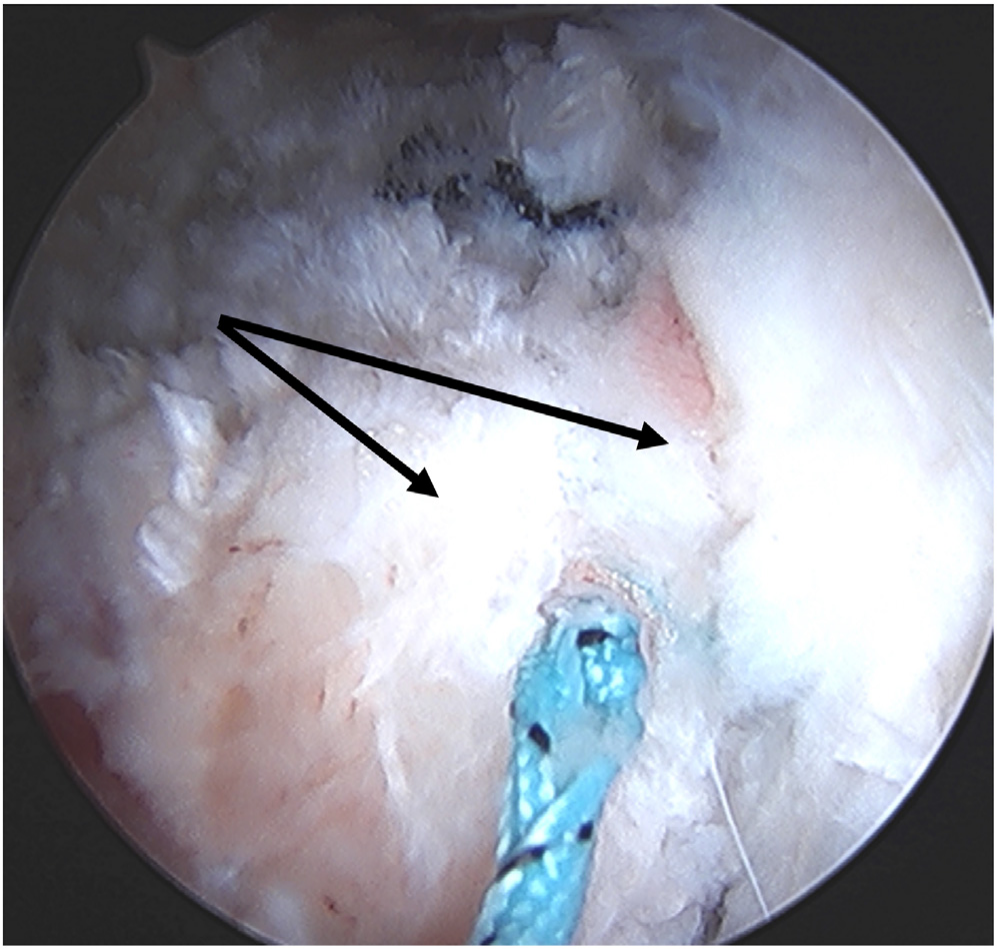
View from the mid-anterior portal with the left hip off traction showing capsulotomy repair (leaflets indicated by black arrows) using nonabsorbable braided sutures in a simple fashion.

With the drill guide still in place, the delivery cannula can be inserted and the stylet removed. The decompression cavity can be irrigated and suctioned if desired. The previously prepared autologous and allogeneic bone graft–contrast mixture can then be gradually injected into the delivery cannula and expelled into the decompression cavity using the stylet. Delivery of the bone graft can be verified visually using fluoroscopy. Once the decompression cavity is filled, additional graft can be placed while withdrawing the delivery cannula to back fill the reamed path (Fig 7). The hip is then distracted again through traction and the arthroscope placed into the joint to confirm no penetration of the femoral head and/or bone graft extravasation. Traction is then released, and the capsulotomy is then repaired using 2 nonabsorbable tape sutures in a simple technique (Fig 8). The wound is then irrigated, and standard closure is performed in a layered fashion.

## Discussion

Because of its insidious onset and progression, often in early adulthood, AVN of the hip is a devastating condition that can have profound, lasting consequences on a patient’s functional status and quality of life. Cases that progress to femoral head collapse predictably progress to end-stage hip arthritis.^1^ Identification of AVN in the precollapse stage is crucial to intervene with treatments to slow its progression. If precollapse AVN is diagnosed and symptomatic, it is generally accepted that operative intervention with core decompression is warranted and may halt progression compared with nonoperative treatments.^1^, ^2^, ^3^^,^^10^

Multiple authors have suggested combining hip arthroscopy with core decompression to address concomitant labral tears or other intra-articular patholog.^4^, ^5^, ^6^, ^7^, ^8^ Arthroscopy allows the surgeon to address assess the femoral head visually while addressing all potential pain generators and arthritic precursors in 1 surgery. Additionally, prior core decompression techniques describe using multiple drill holes or fixed-diameter reamers to obtain decompression. The current technique described allows for targeted decompression of the avascular portion of the femoral head using an expandable reamer. This allows for minimal reaming of the lateral femur and femoral neck while still facilitating thorough debridement of necrotic bone and easy placement of injectable bone graft. In combination with hip arthroscopy, this technique allows for complete treatment of AVN along with associated intra-articular hip pathologies.

## Disclosures

The authors declare the following financial interests/personal relationships which may be considered as potential competing interests: D.W. is a consultant or advisor for Newclip Technics, DePuy Synthes Mitek Sports Medicine, Vericel Corporation, and Cartilage; has received funding grants from Vericel Corporation and Immunis; has received travel reimbursement from Arthrex; and has equity and stocks with Cartilage and Overture Orthopaedics. All other authors (T.R.M., C.E.C.) declare that they have no known competing financial interests or personal relationships that could have appeared to influence the work reported in this paper.

## Supplementary Data

Video 1 After positioning the patient supine on the postless traction table, bone marrow aspirate is collected from the left iliac crest, concentrated, and mixed with AlloSync (Arthrex) and iohexol contrast media. Diagnostic hip arthroscopy is then performed via mid-anterior and anterolateral portals with an interportal capsulotomy and traction applied. Intra-articular pathology is addressed as indicated (labral repair in this case with addition of a distal anterolateral portal) and traction is released. A lateral hip incision is then made to facilitate guidewire placement into the necrotic area of the femoral head under fluoroscopy. The expandable reamer system is then used to ream the necrotic bone, taking care to avoid reaming within the femoral neck. After sufficient decompression, the reamer is withdrawn, and the bone graft mixture is injected into the femoral head and neck. Radiographs are taken to ensure appropriate graft fill without extrusion into the hip joint. Traction can then be reapplied, and the arthroscope is reinserted to assess for graft extrusion. Finally, capsular closure is performed through the anteroalteral portal.
